# Hemorrhagic Vestibular Schwannoma: Case Report and Literature Review of Incidence and Risk Factors

**DOI:** 10.7759/cureus.10183

**Published:** 2020-09-01

**Authors:** Taha Shahbazi, Mohammadmahdi Sabahi, Mahdi Arjipour, Badih Adada, Hamid Borghei-Razavi

**Affiliations:** 1 Neurosurgery Research Group (NRG), Student Research Committee, Hamadan University of Medical Science, Hamadan, IRN; 2 Neurosurgery Research Group (NRG), Student Research Committee, Hamadan University of Medical Sciences, Hamadan, IRN; 3 Neurosurgery, Hamadan University of Medical Science, Hamadan, IRN; 4 Neurosurgery, Cleveland Clinic Florida, Weston, USA; 5 Neurosurgery, Pauline Braathen Neurological Center, Cleveland Clinic Florida, Weston, USA

**Keywords:** intratumoral hemorrhage, subarachnoid hemorrhage, vestibular schwannoma, acoustic neuroma, hemorrhage

## Abstract

Hemorrhagic vestibular schwannoma (HVS) consisting of acute intratumoral and subarachnoid hemorrhage presents with acute nausea, vomiting, facial numbness, headache, loss of consciousness, and significant functional impairment of the facial and vestibulocochlear nerves. The current case is of a 31-year-old man who was presented with acute left lateral suboccipital headache, vomiting, ataxia, and loss of consciousness. Brain CT revealed a large iso-intense lesion with internal hematoma at the left cerebellopontine angle in association with internal acoustic canal dilation. In addition, MRI confirmed a 32 x 25 x 26 mm vestibular schwannoma (VS) with 20 x 15 x 5 mm intratumoral hematoma. The patient had undergone left lateral suboccipital craniotomy and microscopic tumor resection. Pathological findings revealed that his lesions were VS. The average incidence of HVS is around 2.15 cases per year worldwide. Therefore, HVS incidence in proportion to VS is very low and consequently rare.

## Introduction

Vestibular schwannoma (VS), also called acoustic neuroma, is a benign tumor of the vestibulocochlear nerve that has an incidence of 11-13 cases per million every year. It arises from Schwann cells at the Obersteiner-Redlich zone [1-3].

Hemorrhagic VS (HVS) is a rare phenomenon. Only limited cases are reported and there has been no definite estimation about HVS incidence until now. When significant HVS occurs, patients may experience acute neurological defects [4]. Considered risk factors include huge tumor size, cystic development, hypointense parts in T2-weighted magnetic resonance (MR) signal, hemosiderin deposition due to intratumoral hemorrhage (ITH), and a history of anticoagulant therapy [5,6].

The treatment is based on surgery, and radiation therapy (RT) and observation are other choices of treatment. There are three standard surgical approaches: retromastoid suboccipital (retrosigmoid), translabyrinthine, and middle fossa approach (MFA). Although surgery is the treatment of choice for VS, there are many different RT techniques including single-session stereotactic radiosurgery (SRS), fractionated conventional RT, fractionated stereotactic RT (FSRT), and proton therapy [7-10].

Herein, we report a case of a patient who was admitted with HVS. Some similar cases have also been reviewed as literature review.

## Case presentation

All procedures performed in studies involving human participants were in accordance with the ethical standards (code: IR.UMSHA.REC.1399.388) and the 1964 Helsinki Declaration and its later amendments or comparable ethical standards. Of note, informed consent was obtained from the patient in order to use his information for the case presentation. A 31-year-old shepherd man was referred to our center due to loss of consciousness. He was able to communicate and explained his experiences of a sudden severe headache in the left lateral suboccipital (retroauricular) region, as well as left hemi-facial numbness, nausea, and vomiting. After a while, he developed disequilibrium and loss of consciousness instantly after walking a few steps. Neurological examination showed he had facial paresis (House-Brackmann grade II), vertigo, and ataxia, whereas other examinations were normal.

His past medical history revealed he had a history of appendectomy 30 months before current admission. He had also developed deep vein thrombosis and pulmonary emboli 18 months ago and had used aspirin after thrombolytic therapy. Moreover, he had a history of surgical removal of lung hydatid cyst 12 months ago as well as a history of headache for three years, which was intractable to any treatment. His prothrombin time (PT), partial thromboplastin time (PTT), and international normalized ratio (INR) were normal, and no abnormal findings were reported in lab results.

**Figure 1:**
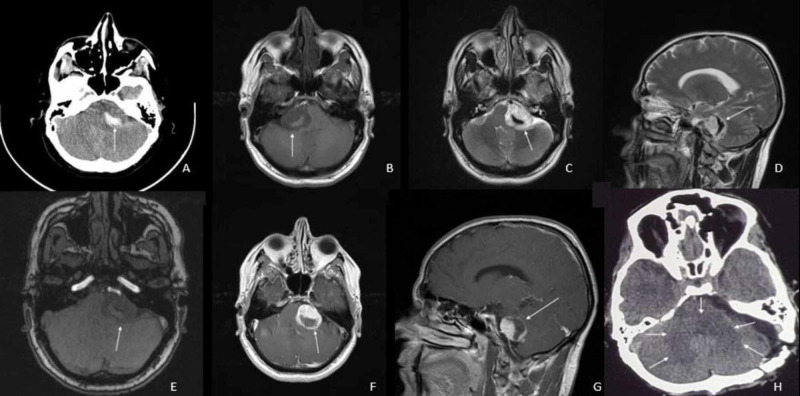
Patient's Preoperative Imaging (A) Brain CT shows an extra-axial lesion at the left CPA region with intralesion hematoma. (B and C) Axial T1- and T2 -weighted images, respectively, show extra-axial lesion, which was hypointense on T1-weighted images and hyperintense on T2-weighted images. Intralesional hematoma is iso- and hypointense on T1- and T2-weighted images, respectively. (D) Sagittal T2-weighted image. (E) Axial MRI source image. (F and G) Contrast T1-weighted axial and sagittal images show homogenous avid tumor enhancement. (H) Contrast CT show postoperation changes and tumor resection with retrosigmoid approach.

Brain CT revealed extra-axial lesion at the left cerebellopontine angle (CPA) region with an intralesion hematoma that expanded tumor volume (Figure 1A). The left internal acoustic canal (IAC) was dilated in comparison with the right side. The brain stem and fourth ventricle were compressed with the mass effect of hemorrhagic tumor. There was some ventriculomegaly but no periventricular edema and hydrocephalus.

In the MRI, 32 x 25 x 26 mm VS at the left CPA with extension to IAC was seen. The lesion was hypo- and hyperintense on T1-weighted image (T1WI) and T2-weighted image (T2WI), respectively (Figures 1B-1D) and enhanced dens homogenous on contrast T1WI (Figures 1F, 1G). Intralesion hematoma measuring 20 x 15 x 5 mm was iso- and hypointense on T1WI and T2WI, respectively, without T1WI enhancement contrast (Figure 1).

**Figure 2:**
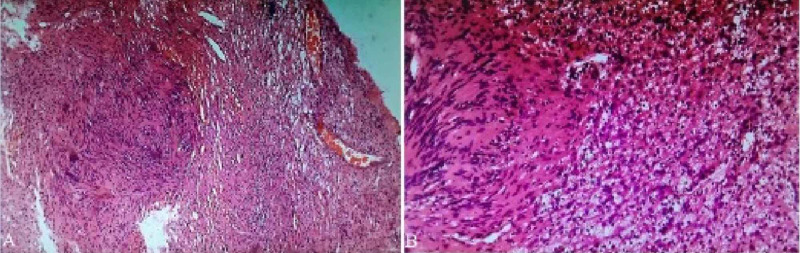
Lesion Microscopic View (A) Low power field. (B) High power field microscopic view shows vestibular schwannoma. Antoni A and B patterns are seen in the collagenous background. In the hypercellular Antoni A areas, intersecting fascicles consisting of spindle cells with buckled nuclei are seen. These cells form Verocay bodies with nuclear palisading, making alternating bands of nuclear and anuclear areas. In the hypocellular Antoni B areas, the prominent myxoid extracellular matrix takes the spindle cells apart.

The patient was operated at semi-lateral supine position with left lateral suboccipital craniotomy and microscopic retrosigmoid approach. Intratumoral hematoma was seen during tumor resection. The tumor was totally resected, and the facial nerve was preserved. The patient’s postoperation course was uneventful and without any new neurological deficit (Figures 2A, 2B).

In the hematoxylin and eosin (H & E) staining, hypercellular (Antoni A) and hypocellular (Antoni B) areas are seen. Dense Antoni A areas consisting of interlacing bundles of spindle cells with oval nuclei, eosinophilic cytoplasm, and indistinct cytoplasmic borders are also seen. Hypocellular dense Antoni B areas are composed of haphazardly arranged spindle cells in loose myxoid collagen fibers. Blood vessels with hyalinized walls were visible.

## Discussion

The VS is an uncommon intracranial tumor and frequently presents with chronic hearing loss, headache, tinnitus, disequilibrium, and facial numbness, whereas acute overt hemorrhage including subarachnoid hemorrhage (SAH) and ITH as its first presentation is quite rare [11].

Of all intracranial hemorrhages, 1-11% belong to hemorrhagic brain tumors. SAH that arises from brain tumors accounts for 0.4% of all cases of SAH. Furthermore, 1.7- 10% of brain tumors cause intracerebral hemorrhage [12]. ITH generally occurs in 11% of all cranial tumors, and its occurrence in glioblastoma multiforme, pituitary adenomas, choriocarcinomas, oligodendrogliomas, choroid plexus papillomas, and meningiomas is also prevalent.

According to Mathkour et al.’s literature review, there were only 48 cases of VS secondary to ITH, whereas in accordance with our review of the literature (Table 1), there were 97 HVS cases since 1974 including our case [13].

Based on the reported cases, the average incidence of HVS worldwide is approximately only 2.15 cases per year; therefore, HVS is a quite rare entity among VS tumors.

Some studies have reported hemorrhagic cystic VS (CVS) and therefore it can be considered as a risk factor for HVS (Table 1). Although our case’s tumor was not of cystic type, CVS has been characterized with faster expansion rate than the solid ones, rapid nerve involvement, and development of variable symptoms. CVS is more commonly demonstrated with fluid-fluid levels and hemosiderin deposition on imaging. Moreover, it has been argued that ITH can result in the formation of the cyst [14].

**Table 1:** Reported Hemorrhagic Vestibular Schwannoma Abbreviations: AF, arterial fibrillation; CABG, coronary artery bypass grafting; CAD, coronary artery disease; CN, cranial nerve; CSF, cerebrospinal fluid; EVD, external ventricular drain; F, female; GFAP, glial fibrillary acidic protein; HB, House-Brackmann grade; HD, hearing disturbance; HTN, hypertension; ITH, intratumoral hemorrhage; IVH, intraventricular hemorrhage; LAFT, lack of access to full text; M, male; MI, myocardial infarction; NR, not reported; NS, not otherwise specified; N/V, nausea/vomiting; PMH, past medical history; POC, postoperative conditions; RF, risk factor; RM, retromastoid; RS, retrosigmoid; SAH, subarachnoid hemorrhage; SO, suboccipital craniotomy; SRS, stereotactic radiosurgery; TBI, traumatic brain injury; TL, translabyrinthine; VPS, ventriculoperitoneal shunt; VS, vestibular schwannoma

Remarks	Outcome, recurrence	Tumor characteristics	Treatment	Pre/postsurgery HB grade	SAH	Size (cm)	Clinical presentation	Age(years)/Sex	Case number
History of HTN and pregnancy	Death	ITH/macroscopic characteristics: gray, firm, and not attached to the tentorium	Conservative	NR/NR	Yes	2 x 3 x 3.5	N/V, facial hemiparesis, progressive slowed mental reactions	64/F	1
Bloody CSF	Good, NR	ITH	Elective Surgery	NR/NR	Yes	Large	Headache, N/V, vertigo	21/F	2
POC: persistent ataxia, facial paresis, and HD	Good, NR	No ITH	SO	NR/NR	Yes	4	Headache, HD, facial numbness and paresthesia, nystagmus, decrease corneal reflex, hypoesthesia and hypoalgesia of left trigeminal nerve	54/F	3
NR	Death	No ITH	Conservative	NR/NR	Yes	NR	NR	64/M	4
LAFT	Good, NR	No ITH	LAFT	NR/NR	Yes	NR	Headache, ataxia	54/M	5
Hydrocephalus and VPS	Death,	ITH	Surgery NS	NR/NR	No	NR	Ataxia, facial hemiparesis, headache, hydrocephalus, altered mental status	71/F	6
PMH: deafness, ataxia and sensory change over the left trigeminal territory; job: farmer	Good, NR	No ITH	Surgery NS	NR/NR	Yes	3	Ataxia, HD, facial hypoesthesia	66/M	7
Xanthochromic CSF; PMH: tinnitus for 2 years, unsteadiness of balance with vertigo/no facial palsy	Good, NR	ITH	SO	NR/NR canal.	Yes	4	Headache, nystagmus, dizziness, otalgia, N/V, HD, absent ice-water caloric response, decreased corneal reflex, decreased right external auditory sensation	61/M	8
Subdural hemorrhage	Good, NR	ITH	SO	NR/NR	Yes	5	Ataxia, facial hypoesthesia, facial hemiparesis, headache, N/V, papilledema	49/M	9
RF: pregnancy	Good, NR	ITH	LAFT	NR/NR	No	NR	Headache, dizziness, swallowing difficulty	33/F	10
LAFT	Good, NR	ITH	LAFT	NR/NR	Yes	NR	Tinnitus, HD	54/M	11
NR	Good, NR	ITH	Surgery NS	NR/NR	NR	4	Headache, HD, tinnitus	33/F	12
LAFT	Good, NR	No ITH	LAFT	NR/NR	Yes	NR	Headache, N/V	32/M	13
LAFT	Good, NR	ITH	LAFT	NR/NR	Yes	3	Headache	61/F	14
Anticoagulation valve replacement	Death	ITH	LAFT	LAFT	LAFT	Large	Headache, diplopia, facial hypoesthesia, unsteadiness	58/M	15
RF: heavy weight lifting, head injury; POC: facial nerve was preserved.	Good, NR	ITH/no cystic tumor	SO, TL	NR/NR	Yes	3.2	Headache, HD, facial hemiparesis, otalgia, N/V, vertigo, sweating, nystagmus, taste perception was absent on the left side	19/M	16
Physical exercise/PMH: Grave's disease, HD, and tinnitus	Good, NR	ITH	Surgery	NR/NR	Yes	NR	Headache, N/V, facial sensory change, loss of consciousness, stiff neck, Brun's nystagmus, absence of right corneal reflex	47/F	17
NR	Good, NR	ITH	RS	NR/NR	No	NR	Facial sensory change, HD	37/F	18
RF: HTN	Good, NR	ITH	SO	NR/NR	No	NR	Headache, N/V, diplopia	60/M	19
NR	Good, NR	ITH	NR	NR/NR	No	5	Headache, HD, vomiting, diplopia, facial paresthesia, nystagmus	42/M	20
Headache, cysts filled with dark red or xanthochromic fluid	Good, NR	ITH, cystic tumor	Surgery NS	NR/II	No	5	Headache, HD, ataxia, trigeminal and facial nerve palsies	56/M	21
NR	Good, NR	ITH	Surgery	NR/NR	No	3	Headache, tinnitus, HD	39/M	22
Postoperation facial weakness and HD	Good, NR	ITH	SO	NR/NR	No	4	Ataxia, dizziness, headache, N/V, vertigo, nystagmus, decreased corneal reflex, stiff neck	65/F	23
Minor head injury; after surgery, slight left facial weakness and hearing loss persisted	Good, NR	ITH/tumor had multiple cysts which consisted of xanthochromic and dark brown elements	SO	NR/NR	No	3	Ataxia, facial hemiparesis, HD, Brun’s nystagmus	62/F	24
NR	Good, NR	ITH	NR	NR/NR	No	5.4	Ataxia, HA, HD	41/M	25
NR	NR, NR	NR	SO	NR/NR	Yes	NR	Gait disturbance, Headache HD, N/V, facial numbness	70/M	26
NR	NR, NR	ITH	SO	NR/NR	Yes	3	Progressively worsening left suboccipital neck pain, ataxia, facial paresis	42/F	27
Gag reflexes were depressed, no facial weakness or sensory loss was present	NR, NR	ITH	Surgery	NR/NR	No	2,3	HD, increasing gait Instability, dizziness	66/F	28
NR	NR, NR	NR	Surgery	NR/NR	Yes	4	Severe neurological impairment due to cerebral compression, persistent diplopia, headache, HD	23/M	29
PMH: persistent left tinnitus and left hearing loss, feeling of tingling appeared around the left tongue and left lip, vertigo	Good, NR	ITH	Posterior subcraniotomy	NR/NR	No	4 x 3 x 3	HD, ataxia, unilateral headache and trigeminal symptom, facial paresthesia, decreased perception of the third branch (only around the lips), decreased taste sensation	46/F	30
NR	Good, NR	ITH	RS	NR/NR	No	3	Ataxia, facial hemiparesis, HD, N/V	63/F	31
NR	Good, NR	ITH	RS	NR/NR	No	3.8	Ataxia, headache, HD, N/V	45/F	32
NR	Good, NR	ITH	RS	NR/NR	No	2.8	Facial hypoesthesia, headache, HD	31/M	33
NR	Good, NR	ITH	RS	NR/NR	No	3	Ataxia, headache, HD	54/M	34
NR	Good, NR	NR	RS	NR/NR		3	Headache, nystagmus	28/M	35
CN VII to XII paralysis	Good, NR	ITH	LAFT	LAFT	LAFT	2.6	Tinnitus, ataxia, facial paralysis, hoarseness	65/F	36
NR	NR, NR	ITH	NR	NR/NR	No	NR	HD, dizziness, absent gag and left corneal reflexes	NR	37
NR	Good, NR	NR	Surgery	NR/NR	No	Large tumor (>2 cm)	HD, facial hemiparesis, cerebellar symptomatology, N/V	70/M	38
None of the other five patients (41-45) had an acute onset; none of the patients had systemic HTN	Good, NR	ITH/cystic tumor	RM	NR/NR	No	3	Tinnitus, HD, headache, facial paresis, ataxia	65/F	39
	Death	ITH/cystic tumor	RM	NR/NR	Yes	4.2	NR	35/F	40
	Good, NR	ITH/cystic tumor	RM	NR/NR	No	4.0	NR	30/F	41
	Good, NR	ITH/cystic tumor	RM	NR/NR	No	6.0	NR	32/M	42
	Good, NR	ITH/cystic tumor	RM	NR/NR	No	5.0	NR	43/F	43
	Good, NR	ITH/cystic tumor	RM	NR/NR	No	3.8	NR	72/M	44
	Good, NR	ITH/cystic tumor	RM	NR/NR	No	5.2	NR	44/F	45
PMH: HD on the left and tinnitus for 20 years and headache from 1 week prior to admission	Good, NR	ITH	SO	NR/NR	No	3.5	Left HD, gaze nystagmus toward the right	66/F	46
Aspirin for his pain	Good, NR	ITH	SO	NR/NR		2.5	HD, intermittent tinnitus, mild intermittent left-sided headaches	36/M	47
No tumor recurrence after 6 years	Good, No	ITH	SO	NR/II	No	4	Ataxia, facial hypoesthesia, Headache, HD, N/V, tinnitus, hydrocephalus	35/F	48
NR	Good, No	ITH	RS	NR/NR		NR	HD, headache, tinnitus, no caloric response	25/F	49
PMH: HTN, hypercholesterolemia	Good, No	ITH	TL	NR/NR	No	1.9	Dizziness, HD, N/V, tinnitus	55/M	50
NR	Good, No	ITH/cystic tumor	RS	VI/VI	No	2	Facial hemiparesis, HD	73/F	51
NR	Good, No	ITH	TL	I/I	No	2.6	Diplopia, facial hypoesthesia, Headache, HD, N/V, otalgia	52/F	52
NR	Good, No	ITH/cystic tumor	TL/RS	NR/NR	No	3.6	Ataxia, facial hypoesthesia, Headache, HD, otalgia	41/F	53
Recurrent IVH and SAH	Good, No	NR	NR	NR/NR	Yes	1	Headache facial hemiparesis, N/V, neck pain	15/F	54
LAFT	Good, NR	ITH/hypervascularized tumor	LAFT	LAFT	LAFT	LAFT	Several CN palsies, Decreased level of consciousness	38/M	55
NR	NR, NR	ITH/cystic tumor	Surgery NS	NR/NR	NR	4.4	HD, gait disturbance	19/M	56
NR	NR, NR	ITH/cystic tumor	Surgery NS	NR/NR	NR	4.5	HD, headache, gait disturbance	64/F	57
Oral anticoagulation treatment	Good, NR	ITH/positive S-100 protein	Surgery NS	NR/NR	Yes	1.5	Dizziness, facial hemiparesis, headache, HD, vertigo, N/V	49/M	58
PMH: dizziness, HTN, and AF treated by calcium channel blocker and anti-vitamin K were reported	Death	ITH	None	NR/NR	NR	3	Diplopia, dysphonia, facial hemiparesis, Headache, HD, CN III, V, VI, VII, IX, X, XI deficit	73/F	59
LAFT	Good, NR	LAFT	LAFT	NR/NR	No	NR	Headache, HD, facial hemiparesis	68/M	60
PMH: chronic right-sided deafness, right facial nerve paresis, and mild ataxia treated using low-dose aspirin	Good, NR	ITH	SRS	NR/NR	No	2	HD, facial palsy	55/M	61
PMH: stereotactic radiation therapy for VS due to her cardiac condition	Death	ITH/cystic tumor/highly vascularized tumor	surgery	NR/NR	NR	NR	Hemiparesis, loss of consciousness	72/F	62
NR	Good, NR	No ITH/no cystic tumor	RS	NR/NR	NR	1.6	HD	41/F	63
NR	Good, NR	ITH/cystic tumor	RS	I/NR	NR	3	Facial hypoesthesia, headache, N/V	48/F	64
NR	Good, NR	No cystic tumor	TL	II/NR	NR	3.6	Ataxia, headache	47/F	65
NR	Good, NR	Cystic tumor	RS	II/NR	NR	4	Dizziness, headache, N/V	26/F	66
NR	Good, NR	ITH/no cystic tumor	RS	II/NR	NR	1.8	Facial hemiparesis, headache	68/M	67
NR	Death	No cystic tumor	VPS	I/NR	NR	2.3	Diplopia, headache, neck pain	66/M	68
PMH: rheumatoid arthritis treated with methotrexate (thrombocyte count remained normal)	Good, No	ITH	VPS, RS	NR/V	NR	NR	Ataxia, headache, HD, papilledema, hydrocephalus	59/F	69
NR	Good, NR	No ITH	SO	NR/NR	Yes	NR	HD	18/F	70
NR	Good, NR	ITH	RS	II/V	No	2.5	Headache, vertigo, N/V	66/M	71
PMH: HTN, hyperlipidemia, CAD with MI, and paroxysmal AF, CABG mitral valve replacement, Warfarin, mitral valve replacement, and prostatectomy	Death	ITH/cystic tumor	Conservative	VI/VI	No	3.2	Ataxia, facial hemiparesis, facial hypoesthesia, HD	69/M	72
Hearing did not improve and he developed mild facial paralysis	Good, NR	ITH	RS	III/III	NR	3.1	Ataxia, sudden onset Headache, HD, nausea, tinnitus	15/M	73
Cerebral angiography was normal	Good, NR	ITH	SO	NR/NR	NR	3	Facial hemiparesis, headache, HD	65/F	74
POC: improved hearing; no recurrence	Good, No	ITH	Observation	I/I	NR	0.4	Ataxia, vertigo, tinnitus	83/NR	75
POC: facial nerve sacrifice; no recurrence	Good, No	ITH	Surgery	I/VI	NR	3.5	HD, headache, ataxia	39/NR	76
POC: improved gait, vertigo; no recurrence	Good, No	ITH	Surgery	I/II	NR	2.0	HD, headache, ataxia, vertigo, trigeminal weakness	66/NR	77
POC: improved gait, vertigo, diplopia; no recurrence	Good, No	ITH	Surgery	VI/II	NR	3.5	HD, headache, facial Weakness, vertigo, diplopia	68/NR	78
No recurrence	Good, No	ITH	Surgery	I/NR	NR	3.1	HD, ataxia, headache, N/V, hydrocephalus	72/NR	79
No recurrence	Good, No	ITH	Surgery	VI/NR	NR	2.4	HD, headache, facial Weakness, vertigo, tinnitus	61/NR	80
PMH; HTN, hypercholesterolemia, thrombosis of the right carotid artery, acute MI, total knee replacement, thoracic and lumbar fractures, diabetic retinopathy, aortic valve replacement, CABG, and postoperative AF, extensive medications, which included anti‑coagulants	Death	ITH/positive S-100, and a low mitotic activity (MIB1) in tumor areas, GFAP staining was negative, cystic tumor	RS	VI/NR	No	NR	Facial hemiparesis, headache, HD	76/F	81
RF: HTN	Good, No	ITH/no cystic tumor	RS	NR/II	NR	2.4	Facial hemiparesis, dizziness, Headache, N/V	66/M	82
RF: HTN anticoagulant (INR 2.8)/requiring VPS	Good, No	ITH/no cystic tumor	EVD, then TL	NR/VI	NR	4.2	Ataxia, dizziness, facial hemiparesis, Headache, HD, Hydrocephalus	39/M	83
RF: HTN, anticoagulant (INR 2.3)	Good, No	ITH/cystic tumor	RS	IV/I	NR	3.1	Dizziness, facial hemiparesis, facial hypoesthesia, headache, HD, hydrocephalus	68/M	84
RF: HTN requiring VPS	Good, No	ITH/no cystic tumor	EVD, then RS	NR/V	NR	3.5	Ataxia, dizziness, facial hemiparesis, facial hypoesthesia, headache, HD, hydrocephalus	72/F	85
NR	Good, No	ITH/cystic tumor	TL	IV/IV	NR	2.4	Dizziness, facial hemiparesis, headache, HD	61/M	86
NR	Good, Yes	ITH/no cystic tumor	RS	III/II	Yes	4.1	Ataxia, diplopia, facial hemiparesis, headache, HD, N/V	62/F	87
NR	Good, No	ITH/no cystic tumor	RS	III/II	No	5.1	Headache, HD, N/V, mental deterioration, hydrocephalus	58/F	88
NR	Good, No	ITH/no cystic tumor	RS	II/II	No	3	Ataxia, facial paresis, headache, HD	65/M	89
PMH: HTN	Death	ITH/no cystic tumor	RS	III/II	Yes	3.2	Facial hemiparesis, facial hypoesthesia, HD, hydrocephalus	48/M	90
NR	Good, No	ITH/no cystic tumor	RS	III/II	Yes	5.1	Diplopia, facial hemiparesis, headache, HD, N/V, hydrocephalus	56/M	91
NR	Good, NR	ITH	RS	II/III	No	3.9	HD, facial paralysis, tinnitus	42/F	92
PMH: HTN	Good, NR	ITH	RS	II/III	No	4.3	HD, dizziness, choke, hydrocephalus	71/F	93
PMH: HTN, dyslipidemia, congestive heart failure and AF, taking warfarin, mild TBI; POC: no confusion, no hydrocephalus	Good, No	Intralesional hemorrhage	VPS	IV-VI/II-VI	NR	3	Hyperacusis, confusion spatial and temporal disorientation, facial palsy, mild hydrocephalus	74/M	94
Postoperation persistent ataxia; PMH: Hodgkin’s lymphoma and gradual hearing loss	Good, NR	ITH	NS	IV/IV	NR	3	Facial hemiparesis, facial hypoesthesia, headache, HD, tinnitus, ataxia	30/F	95
Crocodile tears 1 year after surgery PMH: Meniere’s disease	Good, No	ITH	RS	II/I	No	4.1	Facial hemiparesis, facial hypoesthesia, Headache, HD, N/V, rotational nystagmus, dizziness	40/M	96
PMH: headache for 3 years, appendicitis, deep vein thrombosis, hydatid cyst operation	Good, No	ITH/no cystic tumor	RS	II/III	No	3.2	Loss of consciousness, headache, facial numbness, N/V, disequilibrium, facial paresis	31/M	97

It is assumed that anticoagulant therapy could be a potential risk factor for HVS. In accordance with other reports, our case had a history of anticoagulant (aspirin) therapy [15,16].

Our patient had high physical activities and he was a shepherd. The patients reported by others had a history of pregnancy, farming, heavy weight lifting, strenuous exercise, and hypertension [17-20]. As a result, these items can also be considered as potential risk factors for HVS.

## Conclusions

Micro-ITH may happen more commonly in VS, but clinically significant hemorrhage has a very rare occurrence. HVS risk factors consist of huge tumor size, cystic development, history of anticoagulant therapy, and strenuous activities.
